# AI virtual digital human influencers vs. human influencers: the impact of health short videos on older adults' cognition and attitude

**DOI:** 10.3389/fpubh.2026.1805541

**Published:** 2026-06-04

**Authors:** Jinglun Zhang

**Affiliations:** School of Communication, Universiti Sains Malaysia, Gelugor, Penang, Malaysia

**Keywords:** credibility assessment, dual-processing model, health short videos, older adults, virtual digital human

## Abstract

**Introduction:**

This study examines differences between AI Virtual Digital Human (AI-VDH) influencers and physician influencers in terms of recognizability, credibility assessment, and their influence on health cognition and attitudes among older adults in Chinese short-video contexts.

**Methods:**

A mixed-methods approach combining questionnaires (*N* = 101, α = 0.897) and experimental design was employed to investigate how older adults perceive and evaluate AI-VDH and physician-led health science popularization videos.

**Results:**

Older adults generally expressed confidence in distinguishing AI-generated figures from real persons; however, they often struggled to establish stable recognition criteria in highly realistic scenarios and relied on platform prompts to calibrate their judgments. Credibility assessments were influenced by both content accessibility and life experience, while medical credentials and institutional endorsements functioned as important authoritative cues. In AI-VDH scenarios, participants were more sensitive to presentation cues such as voice, facial expressions, and movements, which prompted greater caution. Both video types produced high self-reported improvements in health knowledge and attitudes, although physician-presented videos achieved slightly higher evaluations.

**Discussion:**

This study extends the dual-processing-three-level framework to embodied AI health video contexts by emphasizing the distinction between subjective confidence and actual recognition performance, while incorporating platform labeling into credibility calibration mechanisms. The findings reveal a calibration bias characterized by “high confidence but unstable recognition standards” among older adults in AI-VDH contexts. Platform prompts such as “suspected AI generation” play an important role in credibility calibration. The study provides practical implications for optimizing platform labeling systems, standardizing health science communication content, and developing AI and health literacy interventions for older adults.

## 1 Introduction

China is the world's most rapidly aging nation with the largest older population, making health topics one of the most highly scrutinized issues in contemporary Chinese society (1). Short videos on social media, with their easily digestible content and high interactivity, have attracted a suśbstantial older adult user base, becoming a key gateway for older netizens to access health science popularization videos. Through algorithmic recommendations and personalized content delivery, health knowledge is rapidly and intuitively conveyed to older adult users via short videos, helping them enhance their health awareness (2). With the rapid advancement of artificial intelligence technology, AI-Virtual Digital Humans (VDHs) are emerging as a novel communication medium, finding application across diverse fields. Within social media, they transcend spatial and temporal constraints while overcoming cost limitations, demonstrating significant potential for health communication (3).

Within social media ecosystems, physician influencers strategically showcase diverse lifestyle scenarios to bolster the authenticity of their professional identity and enhance the persuasiveness of their discourse, thereby attracting user engagement and fostering interactive participation. Such influencers often cultivate personas that blend professional authority with personal charisma, embodying dual roles as both “medical experts” and “online celebrities” (4). Medical influencers on social media exert significant influence in disseminating reliable health information, shaping public health perceptions, and engaging with audiences (5, 6). With the rapid advancement of AI technology, the role of physicians in health communication has expanded further, evolving from real influencers to the construction and utilization of virtual avatars. Although virtual avatars can substitute for human content creation and simple interactions, their production technology remains complex and has not seen widespread adoption. AI-VDHs, however, can replace real individuals in content production and basic interactions—reducing reliance on human performers and lowering production costs for standardized content (3).

AI-VDHs can exhibit highly human-like appearances and audiovisual capabilities, enabling real-time interaction with multiple participants simultaneously (3). While live-stream marketing and news broadcasting currently represent the most prevalent domestic applications (7–10), they are equally suitable for explanatory content with relatively fixed structures where viewers do not demand excessive flexibility in video presentation (3). Nevertheless, the application of AI-VDHs in health-related short videos remains exploratory, with notably insufficient research investigating AI-VDHs for health science communication within a health dissemination context. Furthermore, recent studies indicate that older adults still have limited trust in health information generated or facilitated by artificial intelligence; many express skepticism about its accuracy and prefer to consult human clinicians (11, 12). The authoritative packaging of AI-generated content—such as virtual doctors in white coats—blurs the boundaries of information credibility. As increasing numbers of older adults access health information via short videos, a significant trust gap may emerge in disseminating health knowledge through new technologies. Moreover, the misuse of AI may erode older adults' trust in authentic information, ultimately compromising their health judgements (12, 13).

Given that short videos have become a key channel for the public to access health information, and with AI technology increasingly integrated into the content production process, while this enhances information accessibility, it also raises potential risks regarding information credibility and audience trust. Therefore, it is necessary to systematically evaluate the actual effectiveness of AI-VDH in health communication. Although existing studies have separately examined the application and impact of health communication on social media, physician influencers, and AI-generated content, there is a general lack of direct comparisons between the communication effectiveness of AI-VDH and real physicians in the context of health short videos. In particular, there are significant gaps in understanding the cognitive processing and trust-building mechanisms among the older population. Consequently, against the backdrop of China's aging society and the deep integration of digital technology, this study aims to explore the differences in communication effectiveness between AI-VDH influencers and human doctor influencers in health-related short videos on social media. It focuses on analyzing the impact of different presentation styles on the health cognition and attitudes of the older population, thereby addressing the research gap between existing theory and practice.

## 2 Research background and related literature

### 2.1 AI-VDH influencers vs. human influencers in health communication

With the advancement of artificial intelligence technology, AI-generated short videos on health education have gradually emerged on social media, becoming an emerging form of health information dissemination. In this communication ecosystem, health information disseminators primarily fall into two categories: one consists of “human influencers,” such as doctors and health bloggers; the other consists of AI-VDH.

Human influencers typically possess greater professional authority and social influence. Particularly in the field of health communication, information led by doctors or professionals is more likely to be viewed by audiences as a credible source; its credibility stems not only from expertise but also from the trust relationships and parasocial interactions formed through long-term engagement (14). Furthermore, human communicators have advantages in emotional expression, contextual understanding, and complex judgment, making them more persuasive when conveying health information that involves uncertainty (15). However, while human influencers possess both expertise and social influence in the dissemination of health information, their content production relies on individual experiences and subjective judgments, which may lead to inconsistencies in information and scientific accuracy. They also face limitations in their ability to scale and standardize their outreach efforts (16).

In contrast, AI-VDHs, as an emerging medium for health information dissemination, offers unique advantages such as high adaptability, strong interactivity, and the ability to simulate human behavior and characteristics, with the aim of providing the highest degree of realism when interacting with human users (17).

In practical applications, the visible characteristics of AI-VDHs, such as appearance and gender, have been demonstrated to be crucial components of their influence. For instance, research by Xue et al. (10) demonstrated that a virtual female AI news presenter using anthropomorphic voice delivery garnered higher audience perception and persuasiveness. To enhance the authenticity and approachability of information delivery, related research has already realized multiple human-like presentation technologies, including anthropomorphic expression generation and lip-syncing (7). Moreover, the non-verbal interactive capabilities of AI-VDHs—such as vocal intonation, facial expressions, eye contact, and body posture—often directly influence audience viewing willingness and information receptivity (18).

Nevertheless, research demonstrates that real individuals maintain significantly higher social influence when conveying persuasive messages, whereas the impact of AI-VDHs depends on the authenticity of its behavioral responses (19). For instance, when AI-VDHs provide positive listening feedback, they can generate greater trust and reliance than human face-to-face communication (20, 21). This finding suggests that, in health communication, audience trust depends not only on the content of the information itself, but is also influenced by the social presence and interactive experience conveyed by the communicator. Consequently, health-related short videos generated by AI-VDHs, combining dynamic visual presentation, emotionally resonant narratives, and interactive designs tailored to user preferences, are reshaping the paradigm of health knowledge dissemination and exhibiting significant communication potential.

Despite AI-VDH's substantial application potential, challenges remain in replacing healthcare professionals. As Tyson and Zysman (22) note, healthcare practitioners—particularly doctors active on social media—rely on advanced cognitive abilities such as interdisciplinary judgement, interpersonal interaction, and emotional communication in daily short video creation. These qualities remain difficult for AI to fully replicate or substitute. However, some studies offer relatively optimistic perspectives. Stein et al. (23) and Winterstein et al. (24) contend that AI-generated personas can achieve comparable communicative efficacy to human influencers in establishing emotional connections and cultivating quasi-social relationships with audiences. Influ Mark Hub (25) further emphasizes that the value of artificial intelligence lies not in merely imitating human behavior, but in its capacity to amplify human creativity. Consequently, whether AI-VDHs can serve as credible information disseminators and achieve dissemination effects comparable to those of human influencers remains to be determined through further systematic research.

### 2.2 Persuasive effect of health-related short videos and credibility of AI-VDH

With the rise of social media, short-video platforms have become an important channel for disseminating health information. Among new media accounts established by China's top 100 physicians, Douyin accounts for a full 100 percent (26). Historically, health-related short videos attracting older adult audiences primarily fell into three categories: Medical, Wellness, Health News and Information (27). However, it remains to be seen whether health education content generated by AI-VDHs can equally effectively engage older adults and influence their cognition and attitude; this question essentially touches on the persuasive nature of health information in complex communication contexts.

The core of health communication lies in translating complex medical knowledge into easily understandable information, thereby influencing the public's perceptions, attitudes, and behavioral decisions, and ultimately promoting disease prevention and improved health outcomes (26). Therefore, the communicative effectiveness of health-related short videos essentially manifests as a persuasion process, the key to which lies in whether it can prompt individuals to change their attitudes and behaviors (28). However, in this persuasion process, whether the information is accepted and adopted depends largely on the audience's assessment of its credibility (29).

Extensive research indicates that credibility is a core mechanism that influences the acceptance of information and the effectiveness of persuasion, sometimes surpassing the impact of the information's logical structure and factual accuracy (30). In the social media environment, users typically form an overall judgment of trust by comprehensively evaluating both the source credibility and the message credibility (31). Therefore, in the dissemination of health-related short videos, the communicator's identity, manner of expression, and content presentation all collectively influence the audience's perception of the information's credibility, which in turn affects the persuasiveness of the message.

Within this framework, AI-VDH, as an emerging information disseminator, exhibits distinct mechanisms for establishing credibility compared to human influencers. On the one hand, research indicates that older adults tend to place greater trust in the knowledge and experience of doctors and medical professionals; particularly when it comes to medical interventions and health-related decisions, such expert sources are viewed as more reliable (12). On the other hand, research also indicates that virtual technologies play a positive role in health management and health education; for example, virtual guidance in short videos can enhance health awareness and promote more active lifestyles (32, 33). Furthermore, in certain utility-oriented contexts, AI-generated characters may even be more persuasive than human influencers (34). This reflects a certain contradiction in audiences' trust judgments between relying on the functional value of AI and prioritizing professional medical knowledge, which constitutes a core challenge that AI-VDH may address to achieve effective persuasion in health-related short videos.

Furthermore, as a highly visual and contextual form of communication, audiences' judgments about the credibility of information depend not only on the content itself but are also significantly influenced by non-content-related cues. For example, the presenter's appearance, tone, delivery style, and overall presentation significantly influence the audience's first impressions and trust judgments (35). In this process, the anthropomorphism of AI-VDHs continues to improve, making them increasingly similar to real people in appearance and expression (36, 37), thereby potentially blurring the audience's distinction between “artificial” and “real.” This blurring of boundaries may, on the one hand, enhance the persuasive effectiveness of AI-VDHs, while on the other hand, it complicates the audience's assessment of the credibility of the information source.

Overall, the persuasive effectiveness of health-related short videos depends largely on the mechanisms underlying the formation of information credibility, and the introduction of AI-VDHs further complicates these mechanisms. Particularly among older adults—a demographic with unique information needs and usage habits—there remains a lack of systematic empirical research on whether AI-VDHs can be perceived as a credible information source and whether it can achieve effective persuasion in health communication. Therefore, it is necessary to further explore the differences and underlying mechanisms between AI-VDH influencers and human influencers in terms of perceived credibility and persuasive effectiveness within the context of health education short videos.

### 2.3 Dual-processing model of information credibility assessment

This study draws upon the integrated model proposed by Choi et al. (38), which synthesizes the dual-processing model and the unifying framework of credibility assessment to organize the research approach. Metzger's theory (39) posits that the evaluation process comprises three stages: first, during the exposure stage, an individual's motivation interacts with their cognitive abilities to jointly determine which strategy they employ to assess information credibility; Subsequently, during the evaluation stage, individuals with varying levels of motivation and ability may employ heuristic or systematic methods for credibility assessment, or may opt to bypass the evaluation stage entirely; finally, during the judgement stage, individuals form their final credibility judgement based on the strategy employed and the cues attended to during the evaluation stage. Hilligoss and Rieh's theory (40) posits that both the heuristic and central pathways can be further subdivided into three levels: construct, heuristic, and cue. Individuals first define at the construct level “what constitutes credible information” within a specific context. Building upon this, they form cross-contextual judgement rules at the heuristic level. Finally, at the cue level, through interaction with the specific information object, they extract observable cues from its content, source, and presentation to either support or modify these rules. Choi et al. (38) applied this dual-processing, three-level integrated theoretical framework to the generative AI context, examining university students' credibility assessments of AI-generated academic task information. Oluwadamilola A. Lawal further extended Choi's work by identifying credibility factors for young people's engagement with social media influencer content, confirming the theory's applicability. This study employs a “stage × level” structural framework to examine variables closely related to the context in which older adult audiences view health-related short videos.

However, existing dual-processing, three-level integration frameworks have primarily been developed and validated in text-oriented information environments and for younger audiences, with limited attention paid to information processing in the context of multimodal health-related short videos. In particular, these frameworks fail to fully elucidate how the emergence of AI-VDHs as communicators will reshape the configuration and salience of credibility cues. Furthermore, although the framework combines phased processing with a multi-level cue structure, its theoretical explanation of how audience characteristics—such as older adults' cognitive abilities and media usage experience—influence processing pathways and cue weighting remains inadequate. Therefore, in AI-mediated health communication scenarios, it is necessary to extend this framework through contextual adjustments to better capture the credibility evaluation mechanisms of older audiences across different communicators and presentation formats.

Building on these theoretical limitations, existing empirical studies have shown that individuals with different levels of knowledge, cultural backgrounds, and social groups vary in their reliance on heuristic cues and systematic processing during credibility assessments (41–43). Although Chang et al. (35) and Zhou et al. (44) explored older adults' credibility assessments of media health information, current research on this demographic remains limited, primarily focusing on browsing and sharing text-image health content. Systematic investigation is lacking regarding whether health short videos—particularly multimodal content featuring AI-generated virtual doctor avatars—which are rapidly proliferating in China, alter older adults' credibility processing pathways.

In AI-assisted medical and health communication, existing research indicates that audience trust in physicians/virtual physicians is shaped by three primary cues: firstly, professional credentials and occupational symbols (such as titles, white coats, institutional endorsements) reinforce perceptions of expertise and accountability (45, 46); Second, the clarity and comprehensibility of language directly affect how well information is understood (47, 48); Third, non-verbal cues such as facial expressions, vocal naturalness, and body movements of AI-VDHs alter audience assessments of warmth, competence, and authenticity (49–51). These cues are particularly important in the highly anthropomorphic context of AI-VDHs, as they can both enhance a sense of social presence and create a tension between realism and unnaturalness, thereby influencing judgments of trust.

**Figure 1:**
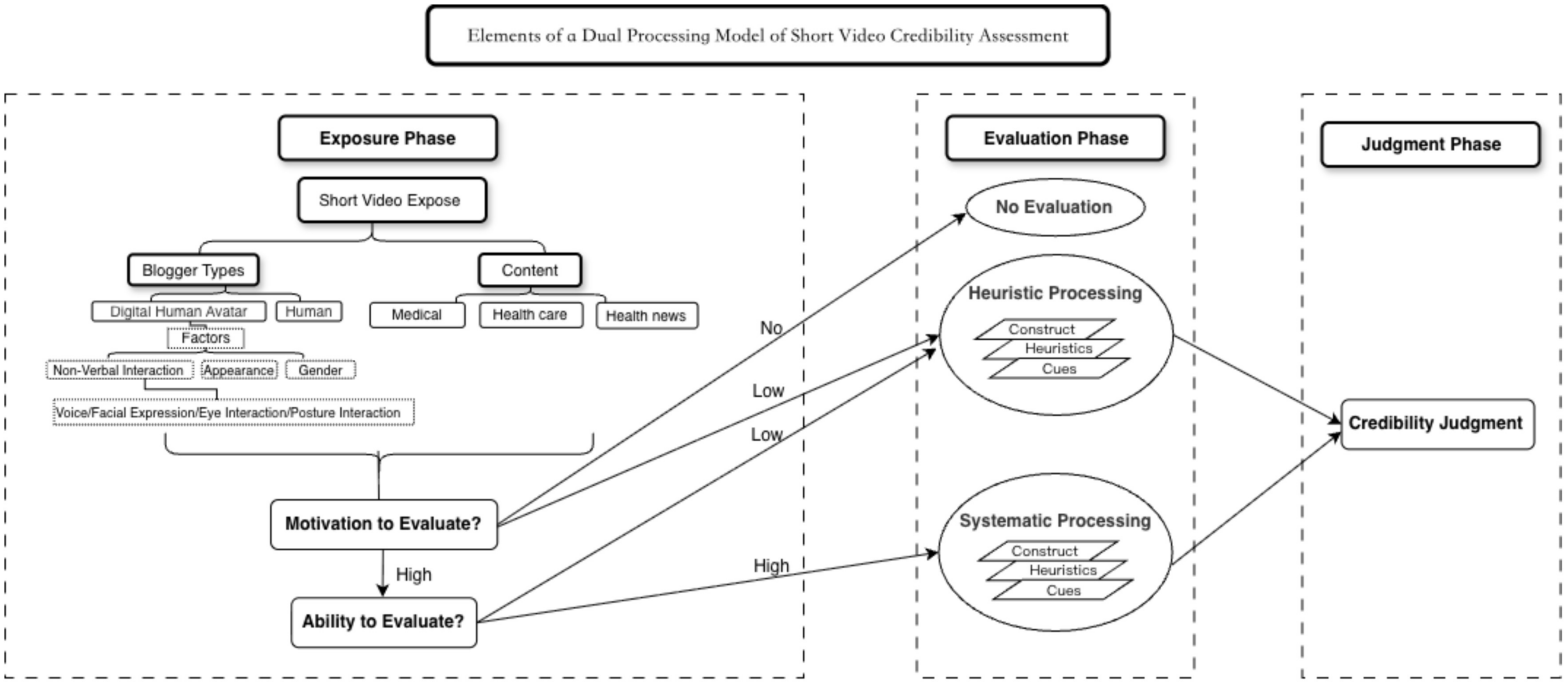
Theoretical framework.

Thus, whilst this study does not encompass all potential motivators and cues within the integrated model, it broadly adheres to the “exposure-evaluation-judgement” process logic. It systematically examines how older adults compare credibility and process information between AI-generated and human-presented health videos across three levels: construct, heuristic, and cue. In summary, the theoretical framework derived from this research is as follows Figure 1.

### 2.4 Health perceptions and attitudes among older adults

Older adults constitute a distinct demographic group with highly concentrated information needs and pronounced cognitive characteristics, representing one of the most critical target audiences in health communication. Their health cognition and attitudinal traits are influenced by physiological, psychological, social, and media factors (52).

Health cognition refers to an individual's understanding, beliefs, attitudes, and perceptions regarding health-related information. For older adults, health cognition is shaped by multiple factors, including declining physiological functions, prior health experiences, sociocultural backgrounds, and their capacity to access and process information (52–55). As individuals age, their cognitive abilities may undergo changes, potentially affecting their comprehension and assimilation of health information (56). Furthermore, some older adults resist using social media due to low digital literacy, viewing technology as unhelpful; however, the majority believe electronic devices can assist them in preventing health deterioration (57).

Secondly, health attitude refers to an individual's evaluative psychological disposition toward health and health-related behaviors; it encompasses positive or negative cognitive, emotional, and behavioral tendencies regarding health. A positive health attitude reflects an individual's strong recognition of the value of health and a psychological inclination to maintain it; it is a key psychological prerequisite that drives individuals to adopt beneficial behaviors and overcome health neglect (58, 59). Consequently, acquiring accurate health knowledge and cultivating a positive health attitude form the foundation for healthy behaviors (60), with greater health skills acquisition correlating to increased likelihood of sustained healthy behaviors over time (61).

However, older adults' limited capacity to evaluate health information constitutes a significant factor in their suboptimal health behaviors (62). This stems from their tendency to rely more on intuition, perception, and emotional cues when assessing digital information sources, rather than systematic reasoning (35). This information processing preference amplifies the role of extrinsic cues—such as the communicator's appearance, vocal tone, and emotional expression—in persuasion processes, thereby heightening the potential impact of AI-VDH within health-focused short videos.

Furthermore, ambivalence toward online healthcare and limited experience with digital tools present additional challenges for older adults' health management (54). Although existing research has explored the application of virtual technologies in older population health management (33), studies specifically examining the impact of AI-generated VDHs in health science popularization videos on older adults' health cognition and attitudes—particularly from a credibility perspective—remain relatively scarce.

Based on the above discussion, the present study proposes the following research questions:

RQ1: Can older adults distinguish between AI-VDH influencers and physician influencers in health short videos?
RQ2: How do the presentation styles of AI-VDH influencers and physician influencers affect older adults' credibility assessments of health information in short videos?
RQ3: How do health short videos delivered by AI-VDH influencers and physician influencers affect older adults' cognition and attitudes toward health information?

## 3 Methods

This study consists of two parts: a questionnaire survey and an experimental study. The questionnaire survey aims to understand participants' perceptions of the impact of health-related short videos on their cognition and attitudes, while the experiment examines their responses under controlled exposure conditions. Together, these two components provide complementary evidence for understanding the impact of health-related short videos. To ensure sample relevance, respondents must be aged 60 or above and have experience viewing at least one AI-generated health short video. This group holds particular research value because they are not only a key target audience in health communication but also a research population of significant theoretical interest. They can help explore how exposure to AI-generated content influences cognitive processing and credibility assessment, thereby providing empirical insights for applying the dual-processing framework in AI-mediated contexts. These screening criteria are designed to ensure that participants have basic exposure to AI-generated content, thereby enabling them to effectively identify and evaluate different informational cues during the experimental tasks and avoid comprehension biases or invalid judgments resulting from a complete lack of relevant experience. Although this criterion may skew the sample toward older adults with higher digital literacy, it can more accurately reveal the cognitive characteristics of “digitally active” older adults in the context of AI-mediated communication.

The questionnaire phase utilized snowball sampling to recruit participants, initiated from a small number of eligible acquaintances and gradually expanding the scope through their social networks; participants for the experimental phase were selected from an older population activity center in a Chinese province, meeting identical criteria. In the experimental phase, eligibility was confirmed prior to participation to ensure that all individuals met the predefined age and prior exposure requirements. Ultimately, 18 participants who satisfied these criteria were included in the experiment. By employing different recruitment methods in the two phases, this study achieved broader coverage in the questionnaire survey while ensuring greater control and consistency in the experimental design, thereby providing complementary evidence for the research objectives.

### 3.1 Questionnaire survey

The questionnaire was collected online via the Credamo platform, with links disseminated through the researcher's personal contacts on WeChat. Screening questions were incorporated to ensure only eligible older adults proceeded, thereby enhancing sample validity and the study's internal validity. The questionnaire (Appendix 1) comprised two sections: Part One gathered demographic information including gender, age, educational attainment, living arrangements, and digital media usage habits; Part Two constituted the main questionnaire, designed according to the study's theoretical framework and covering four core dimensions: (1) Perceptions of previously viewed health-related short videos; (2) Motivations for watching health-related short videos; (3) ability to evaluate health short video content; (4) impact of health short videos on personal health cognition and attitudes.

The primary outcome measures in this study include health cognition, health attitude, behavioral intention, perceived credibility, and information processing patterns. These variables were primarily measured using a five-point Likert scale (1 = strongly disagree, 5 = strongly agree).

The questionnaire data were analyzed using SPSS software, primarily employing descriptive statistics to present sample characteristics and overall trends.

### 3.2 Experimental design

**Figure 2:**
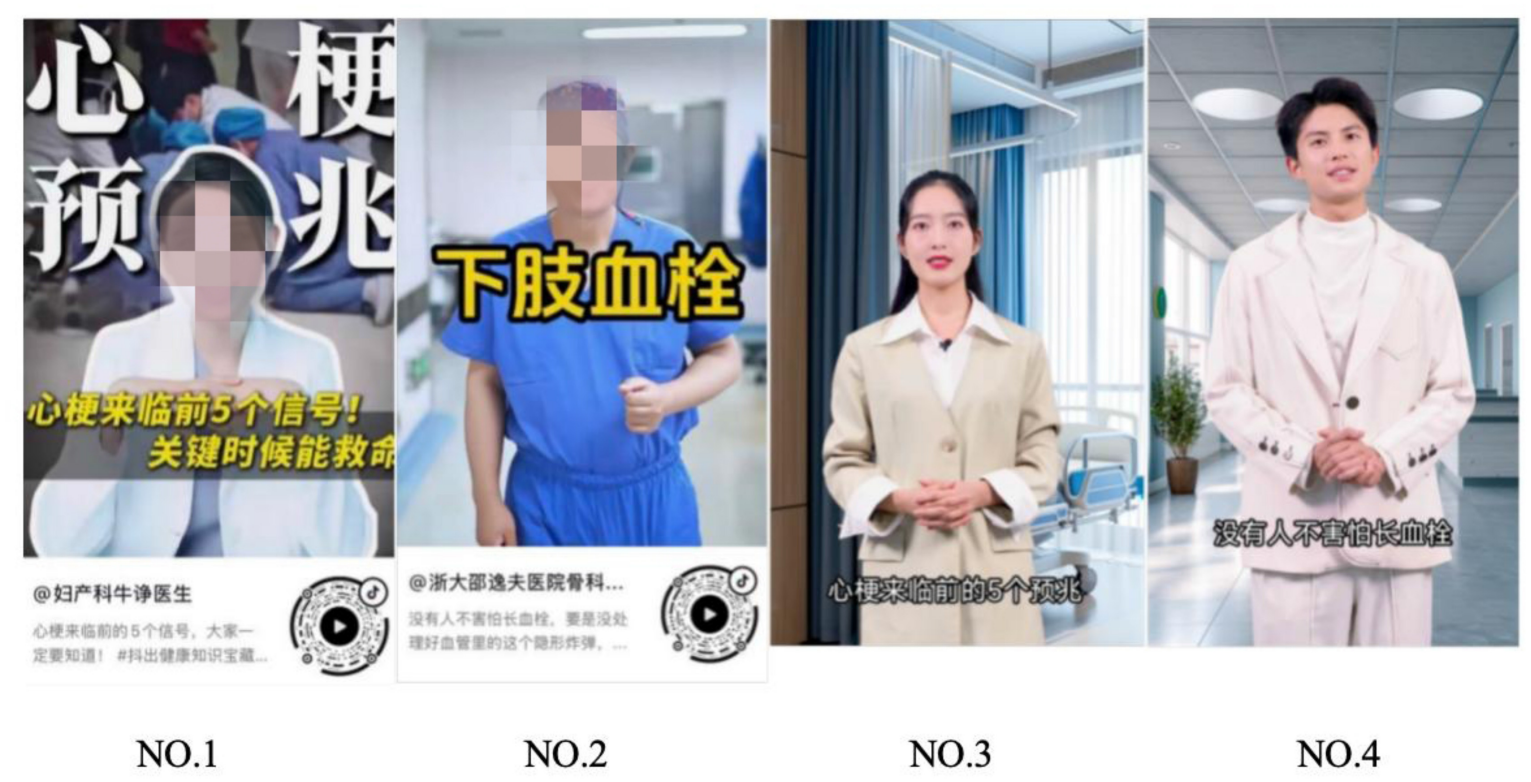
Screenshot from influencers' short video (type 1).

**Figure 3:**
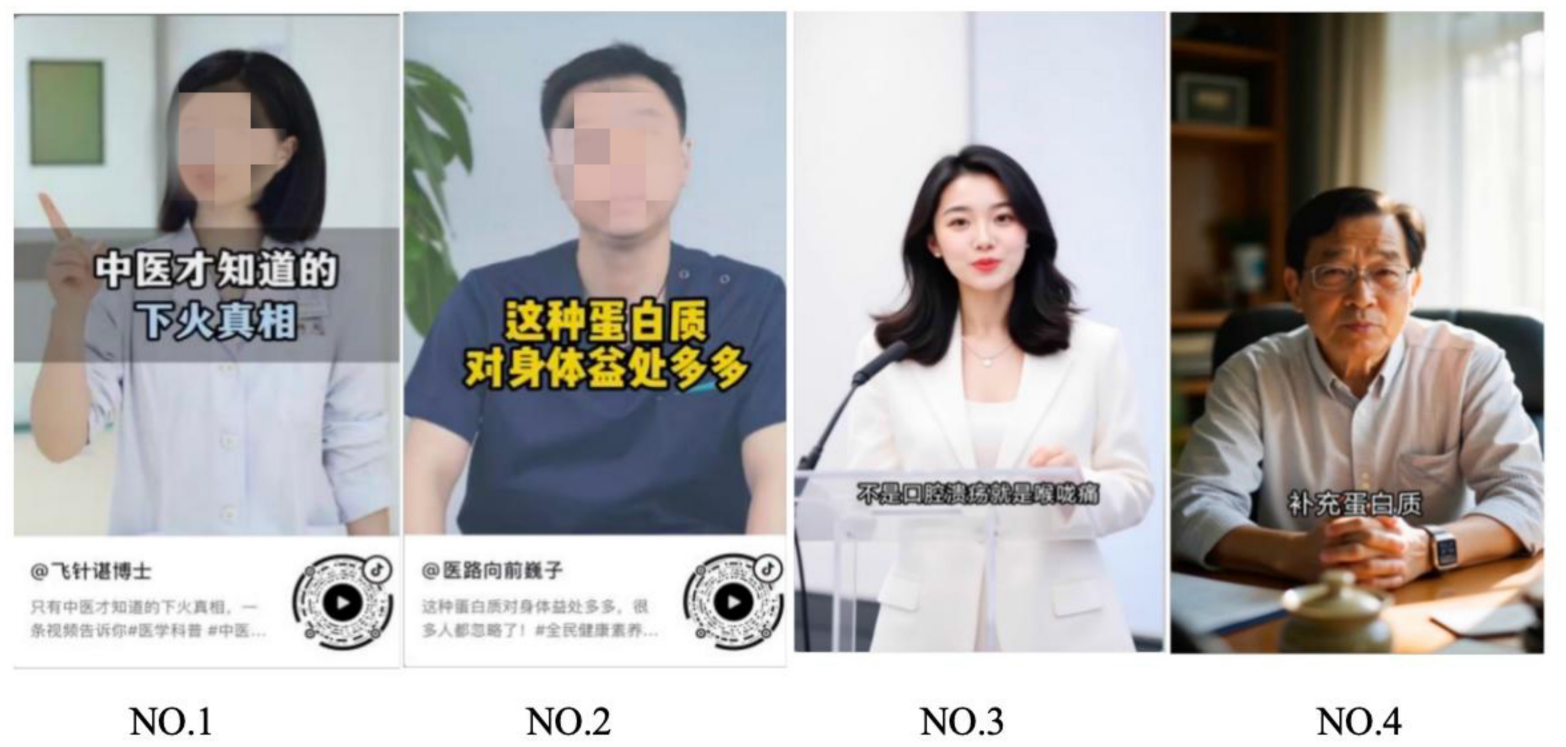
Screenshot from influencers' short video (type 2).

**Figure 4:**
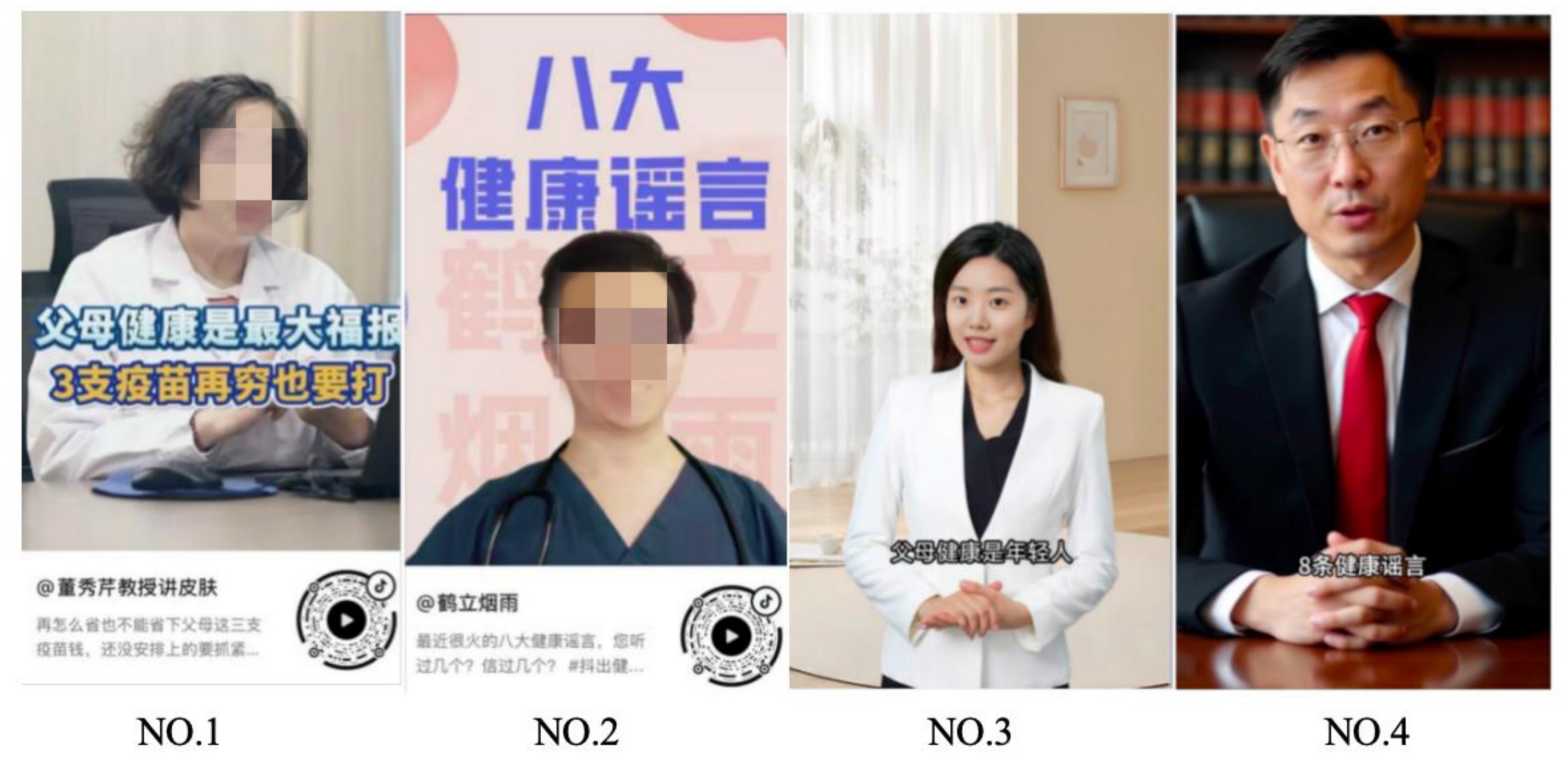
Screenshot from influencers' short video (type 3).

This research focused on Douyin, one of the world's largest short-video platforms. We collected and produced 12 health short videos, each under 3 minin duration, with consistent thematic content across the set (3 themes × 2 videos per theme × 2 influencer types) showed in Tables 1–3 and Figures 2–4, respectively. The physician influencers videos were sourced from Douyin, featuring verified medical professionals with substantial influence, each possessing over one million followers. These videos were selected by researchers based on platform certification. The AI-VDH influencers short videos were produced by ChanJing, with speech content derived from the physician influencers videos. Due to technical limitations of ChanJing, the AI-VDHs did not feature a doctor in a white coat; instead, a white garment was used during production to minimize visual distraction.

**Table 1:** Design plan for the materials of the health short video experiment (type 1).

No.	Influencer type	Account name	Gender	Appearance	Content	Source
Theme 1: medical procedures						
1	Physician influencer	Dr. Niu Jing, Obstetrics and Gynecology	Female	White lab coat	Warning signs of heart attack	TikTok
2	Physician influencer	Dr. Xu Wenbin, Department of Orthopedics, Zhejiang Shao Yifu Hospital	Male	Suit	Thrombosis prevention	TikTok
3	AI-VDH influencer	/	Female	White clothes	Warning signs of heart attack	ChanJing
4	AI-VDH influencer	/	Male	Suit	Thrombosis prevention	ChanJing

**Table 2:** Design plan for the materials of the health short video experiment (type 2).

No.	Influencer type	Account name	Gender	Appearance	Content	Source
Theme 2: wellness and health maintenance						
1	Physician influencer	Dr. Feizhenzhan	Female	White lab coat	Heat-clearing acupoints	TikTok
2	Physician influencer	Weizi, Yi Lu Xiang Qian^*^	Male	Suit	Protein supplementation	TikTok
3	AI-VDH influencer	/	Female	White clothes	Heat-clearing acupoints	ChanJing
4	AI-VDH influencer	/	Male	Suit	Protein supplementation	ChanJing

^*^Yi Lu Xiang Qian means marching forward in medicine.

**Table 3:** Design plan for the materials of the health short video experiment (type 3).

No.	Influencer type	Account name	Gender	Appearance	Content	Source
Theme 3: health news and information						
1	Physician influencer	Professor Cao Xiuqin Talks about Dermatology	Female	White lab coat	Mandatory vaccination for parents	TikTok
2	Physician influencer	He Li Yan Yu^*^	Male	Suit	Science debunks: Drug safety myths	TikTok
3	AI-VDH influencer	/	Female	White clothes	Mandatory vaccination for parents	ChanJing
4	AI-VDH influencer	/	Male	Suit	Science debunks: Drug safety myths	ChanJing

^*^He Li Yan Yu means crane standing in misty rain.

The experiment employed a design combining a control group and experimental groups to examine the impact of different influencer types (AI-VDH influencers and physician influencers) on the cognition and attitudes of older adult participants. Participants were recruited from an older population activity center in a province in China. Given the exploratory nature of this study and the practical limitations in recruiting older adult participants in an offline experimental setting, a total of 18 eligible participants were selected. Participants were assigned to three groups (control group, experimental group 1, and experimental group 2) via simple randomization to ensure balance across groups in terms of demographic characteristics. Although the sample size was small, as an exploratory study, it was sufficient to capture preliminary cognitive responses and information processing patterns in a specific context, thereby laying the groundwork for subsequent large-scale empirical research and the contextual extension of the dual-process, three-level theory.

Both experimental groups were exposed to short videos featuring AI-VDH influencers and medical influencers; the only difference was the order of presentation: Group 1 watched the human-performed videos first, followed by the AI videos, while Group 2 followed the reverse order. This balanced order was designed to control for the potential influence of the primacy effect on the results. The control group did not receive any video intervention during the experimental phase. The specifics of the operation are shown in Table 4.

**Table 4:** Data collection procedure.

Week no.	Activities		Data collection
1	Pre-test for three groups (experimental and controlled group)		Pre-test results
2	Experimental group 1	Experimental group 2	Immediate post-test results
	Physician influencers short video (physician image + platform certification + 3 themes)	AI-VDH influencers short video (virtual image + synthetic voice +3 same themes)	
	Immediate post-test		
	Focus group interview		
Week 3 and 4 (break)			
5	Delayed post-test for three groups		Delayed post-test results

During the first week, all groups completed a pre-test (Appendix 1) to assess their baseline trust and attitudes toward health short videos featuring AI-VDH influencers and human influencers under natural conditions. In the second week, Experimental Group 1 and Experimental Group 2 viewed three health short videos with human influencers and three with AI-VDH influencers, each on different topics, followed by focus group interviews (Appendix 2). Immediate post-tests were administered to participants in both experimental groups. The controlled group did not receive any video intervention and maintained their usual lifestyle to establish a baseline. By comparing the differences between the two groups in the immediate and delayed post-tests, potential confounding factors—such as time effects and changes in the external environment—were successfully eliminated. This design helps to accurately identify the net effects of different video interventions. Subsequently, in Week 5, delayed post-tests (Appendix 3) were conducted for all three groups. Each interview lasted approximately 40 to 60 min and was audio-recorded in its entirety. Before the study, participants provided informed consent, indicating their willingness to participate.

After transcription, the interview data were analyzed using a research question–guided approach to identify key themes that help explain the quantitative findings. The data were organized and categorized accordingly, and key content related to health perceptions, attitudes, and information processing was extracted. Representative statements were selected to provide contextualized interpretation of the quantitative results.

## 4 Results

This study does not focus on comparing user experiences between “AI-VDH and real people,” but instead presents a chain of credibility assessment mechanisms: first, reporting recognition calibration (RQ1)—subjective recognition confidence, post-viewing uncertainty, and the calibration effect of platform prompts; then presenting cue invocation structures (RQ2)—how audiences combine content availability/experience, authoritative credentials, and presentation cues to complete credibility judgments; Finally, it reports judgment outcomes (RQ3)—self-reported changes in health knowledge and attitudes after forming a basic credibility judgment.

### 4.1 Respondent demography

This study collected 101 valid questionnaires. The Cronbach's α coefficient for the questionnaire reliability was 0.897, indicating excellent internal consistency across items and highly reliable data suitable for subsequent analysis. Table 5 presents the demographic data of questionnaire respondents. Overall, the older population—particularly younger seniors aged 60–69—exhibited high activity and persistence in short video usage, with over half spending at least 1 h daily viewing such content. Concurrently, findings reveal that the most frequently viewed health science short video categories among the older adults are Wellness and health maintenance (34.1%) and Medical and Procedures (26.6%), followed by Health News and Information (21.0%) and All that is helpful to me (17.5%). The Rarely Viewed category accounts for merely 0.8%, indicating a high overall viewing frequency predominantly centered on practical content.

**Table 5:** Respondent demography (experiment).

Group	No.	Gender	Age	Time spent on short videos per day	Education level	Types of frequently viewed health education short videos
Controlled group	1	Female	68	1–2 h	High school	B
	2	Female	73	0.5–1 h	High school	ABC
	3	Male	75	0.5–1 h	High school	B
	4	Male	64	1–2 h	Undergraduate	B
	5	Male	60	0.5–1 h	Undergraduate	B
	6	Male	66	0.5–1 h	High school	ABC
Experimental group 1	7	Male	65	0.5–1 h	High school	B
	8	Male	71	0.5–1 h	High school	ABC
	9	Female	60	0.5–1 h	High school	AB
	10	Male	60	0.5–1 h	High school	ABC
	11	Male	83	0.5–1 h	Undergraduate	ABC
	12	Female	67	< 0.5 h	Junior high school	ABC
Experimental group 2	13	Male	64	> 2 h	High school	B
	14	Female	62	0.5–1 h	High school	B
	15	Male	68	< 0.5 h	Junior high school	B
	16	Male	63	>2 h	High school	B
	17	Male	68	< 0.5 h	High school	B
	18	Female	63	< 0.5 h	Undergraduate	B

In the experiment detailed in Table 6, participants ranged in age from 60 to 83 years, comprising 12 males and 6 females. The majority of respondents held a secondary school education. Daily short video viewing time predominantly fell within the “0.5–1 hour” bracket (10 individuals). Respondents primarily consumed health and wellness-themed short videos. Overall, the sample exhibited considerable diversity in terms of gender, age, educational attainment, and video consumption habits.

**Table 6:** Respondent demography (questionnaire).

Gender		Time spent on short videos per day	
Male	50.50%	< 0.5 h	9.90%
		0.5–1 h	28.71%
Female	49.50%	1–2 h	26.73%
		>2 h	34.66%
Age		Education level	
60–69 years	64.36%	Elementary school and below	3.96%
70–79 years	32.67%	Junior high school	11.88%
≥ 80 years	2.97%	High school	33.66%
Undergraduate and above	50.50%
Types of frequently viewed health education short videos			
Medical		26.60%	
Wellness		34.10%	
Health news and information		21%	
All that is helpful to me		17.50%	
Rarely viewed		0.80%	

### 4.2 High confidence and low ability in distinguishing AI-VDH

This section focuses on RQ1, integrating qualitative interviews and quantitative data to explore older adults' ability to distinguish between AI-VDH influencers and human influencers, as well as their cognitive biases. In exploring whether older adult audiences possess the ability to distinguish AI-VDH influencers from real-person influencers in health-related short videos, this study first conducted preliminary exploratory experiments, followed by confirmatory validation of relevant issues through questionnaire surveys. Overall findings indicate that older adults express high confidence in their ability to recognize AI imagery, yet their actual judgements consistently lack systematic identification mechanisms. This gap between perceived ability and actual capability was corroborated by both questionnaire and experimental research.

In the pre-experiment survey, respondents generally expressed high confidence in their recognition abilities. When asked whether they could distinguish between real and AI-VDH influencers in short videos, multiple older participants affirmed they could, emphasizing:

“*Usually, I can tell if it's real or fake with just one look. It's not hard to figure out.” (GA2)*

Some respondents also mentioned having watched AI-VDH livestreams or short videos featuring talking animals, believing AI content still exhibits noticeable visual differences.

“*I've watched the AI version of Liu Qiangdong doing livestream sales — the facial expressions looked kind of stiff.” (GA3)*

However, this confidence was swiftly challenged during the experimental phase. After viewing health-themed short videos featuring a mix of AI and real influencers, most participants admitted in post-test interviews that they struggled to immediately identify the influencer type.

“*If you tell me it's fake, then I'd have to look closely to figure it out.” (GB4)*

Such remarks reveal that when confronted with highly realistic AI digital creators possessing natural, fluent speech, the older adults primary reliance on “experiential judgement” fails, leaving them unable to establish stable, accurate identification criteria.

Furthermore, interviews indicate that some respondents exhibit sensitivity to platform warnings about “suspected AI-generated content.” This suggests that while age may introduce certain technological barriers, many older users possess foundational media literacy, enabling them to make preliminary judgements using platform-provided auxiliary information. In identifying AI content, they do not rely solely on intuition but progressively learn to use platform labels as reference points, demonstrating media adaptability and a willingness to learn.

Building upon this, the quantitative findings provide further corroborating evidence. Q19–Q21 were designed to measure respondents' subjective assessment of their own recognition ability. As shown in Table 7, all three items employed a five-point Likert scale (1 = Strongly Disagree, 5 = Strongly Agree), with all statements phrased positively. Higher scores indicate greater perceived subjective recognition ability. Results indicate that respondents' average recognition ability score was 3.97 with a standard deviation of 0.97. This suggests respondents generally possess strong confidence in their ability to distinguish AI from real humans, consistent with the attitudes observed among older adults in the experiment.

**Table 7:** Respondents' recognition ability.

Questions	Median	Mean	Standard deviation
Q19: I am confident in distinguishing between AI-VDH Influencers and physician influencers in health short videos	4	3.95	0.96
Q20: I can usually easily tell whether a health short video influencers are AI-VDH Influencers or a physician influencers	4	3.84	1.01
Q21: I usually notice the “Potentially AI-generated content, please exercise caution” disclaimer below some short videos	4	4.11	0.95

**Figure 5:**
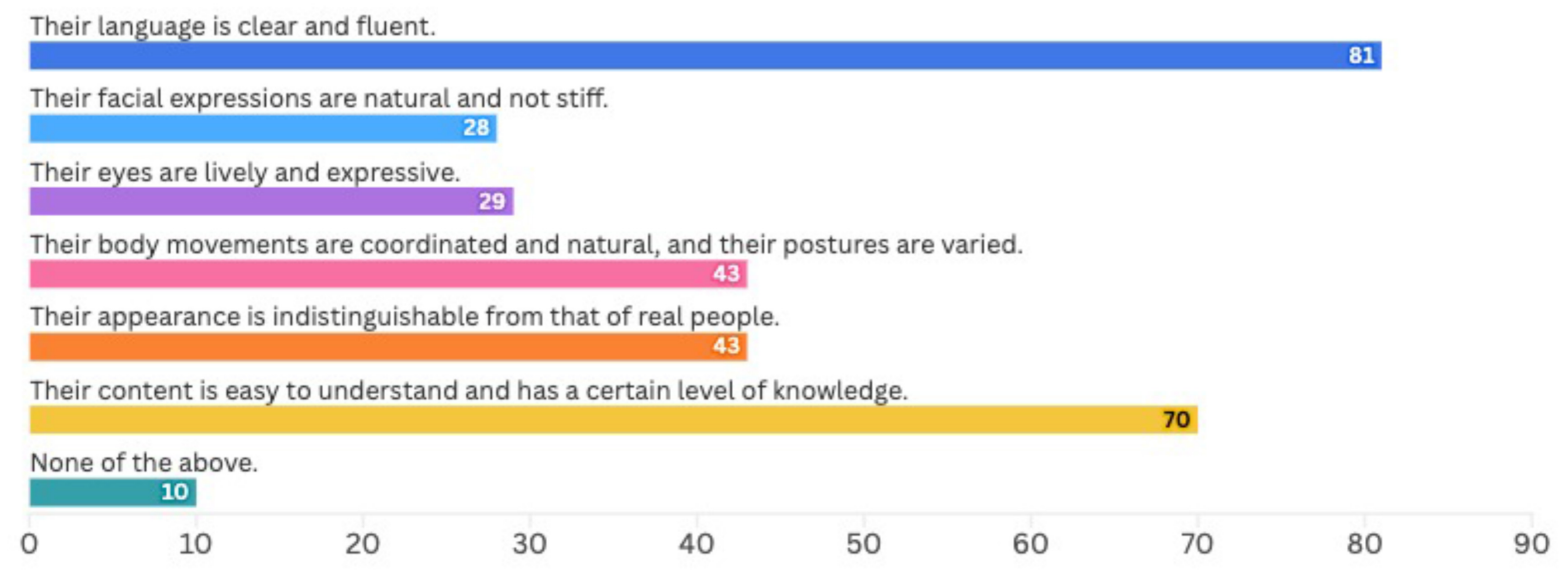
Perceived ratings of older adults on various performance characteristics in health short videos of AI-VDH influencers.

**Figure 6:**
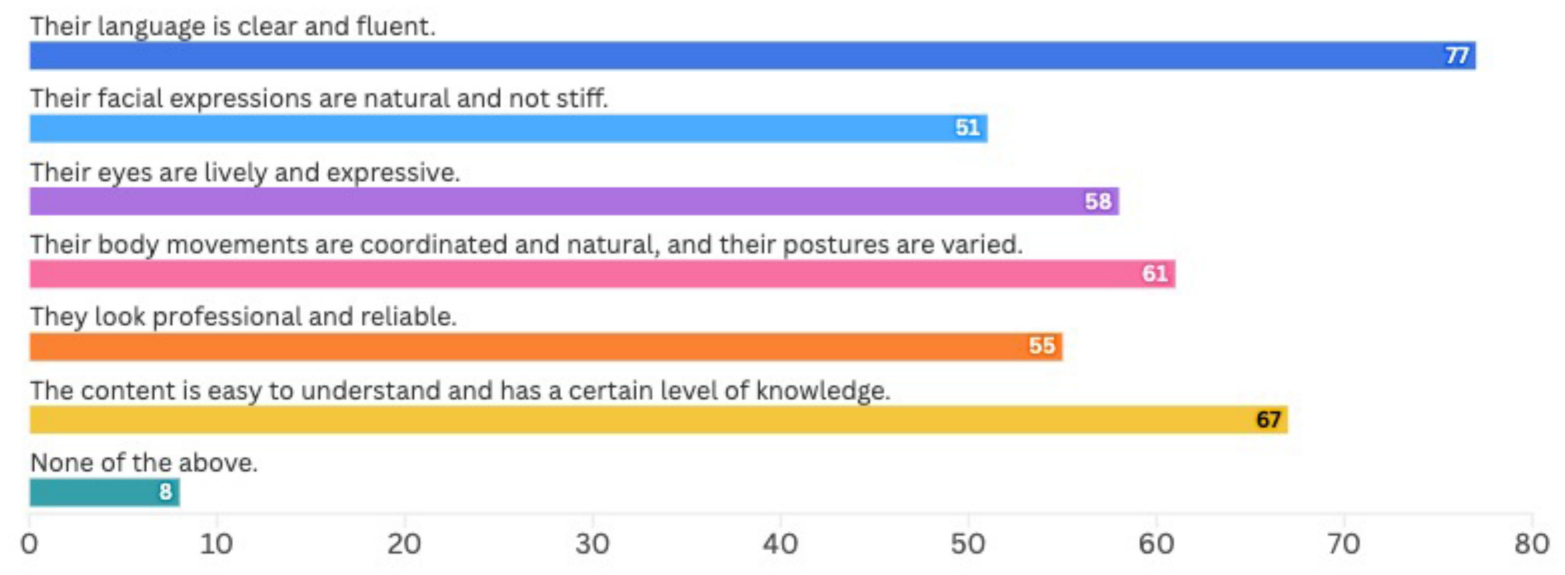
Perceived ratings of older adults on various performance characteristics in health short videos of physician influencers.

Furthermore, as respondents found it difficult to specify distinctions between AI-VDH and human influencers during interviews, this study included two multiple-choice questions regarding “past viewing recognition,” each targeting the presentation characteristics of AI-VDH and human influencers respectively. Results indicate that while both types of influencers received visual recognition, respondents did indeed exhibit differing perceptual emphases in identity judgement (Figures 5, 6).

Regarding AI-VDH influencers, respondents most frequently cited clear language and comprehensible content as positive attributes, indicating that AI-VDH influencers still exhibit perceived weaknesses in local vividness. In contrast, when evaluating human influencers, respondents' judgements relied more heavily on overall impression and perceived consistency.

Comparing the characteristics of both influencer types reveals that while linguistic and content clarity serve as shared credibility indicators, respondents prioritize bodily dynamics, eye contact details, and professional demeanor when assessing authenticity. Conversely, AI-VDH influencer's “realism effect” remains concentrated on linguistic expression and overall lifelike appearance.

### 4.3 Trust disparities under content-driven influence

This section focuses on RQ2, integrating qualitative interviews and quantitative data to analyze the differences in older adults' assessments of the credibility of health information provided by AI-VDH influencers vs. human influencers, as well as the underlying mechanisms. Experimental findings reveal significant dynamic shifts in older adults' trust perceptions toward AI-VDH influencers between pre- and post-tests. During the pre-test phase, notable divergences emerged among participants: most seniors in Experimental Group 1 stated they were unconcerned whether an influencer was AI-generated, prioritizing instead the content's utility and alignment with their personal health experiences.

“*I just care if it makes sense, doesn't matter if it's AI or not.” (GA1)*

In contrast, older adult participants in Experimental Group 2 exhibited pronounced rejection and distrust toward AI, asserting that AI is deceptive and less trustworthy than real people.

“*Even real people lie, let alone AI. I don't trust any of it.” (GB3)*

After viewing the videos and undergoing the experimental intervention, the older adult participants' attitudes toward AI shifted somewhat, as they were unable to accurately distinguish between AI and real people during actual viewing. However, they continued to rely on their own health knowledge and life experience to logically reason about and judge the usefulness of the video content, demonstrating an evaluation stance centered on content and technologically neutral when confronting new technologies.

These findings are further supported by the quantitative results (Table 8). Q14 indicated that most respondents relied more on personal experience and knowledge when evaluating health-related short videos on social media (M = 4.04). Q15 results (M = 3.59) revealed that over half of respondents also made rapid judgements based on external cues such as influencer appearance and the number of likes and comments.

**Table 8:** Respondents' assessment capacity.

Questions	Median	Mean	Standard deviation
Q14: I typically rely on my own experience and knowledge to quickly judge the credibility of health information	4	4.04	0.83
Q15: I often quickly assess the credibility of short video content based on the influencer's superficial image or the number of likes/comments	4	3.59	1.2
Q18: I usually don't specifically evaluate the accuracy of health content in short videos	3	3.12	1.31

Notably, the Q18 findings reveal that some respondents adopt an avoidant processing mode when confronted with complex or highly specialized information. This involves opting out of cognitive processing, neither rationally evaluating content nor making simplistic judgements.

In the experiment, older adults exhibited divergent attitudes toward the AI-VDH influencers. To further compare their trust levels between the AI-VDH influencers and human influencers, the questionnaire included three Likert scale questions (Q23–Q25) (Table 9).

**Table 9:** Respondents' trust in AI-VDH influencers and physician influencers.

Questions	Median	Mean	Standard deviation
Q23: If a influencer is an AI-VDH influencer, I will be more cautious when evaluating health information	4	3.93	1
Q24: I believe physician influencers provide health knowledge that is more professional and useful than AI-VDH influencers	4	3.72	1.21
Q25: I believe physician influencers are more authoritative and trustworthy than AI-VDH influencers	4	3.73	1.15

The findings reveal that despite continuous technological advancements in VDH influencers, human influencers remain subjectively perceived as more credible sources of information.

Furthermore, in examining how different presentation styles of influencers influence older adults' assessment of health information credibility, this study compared respondents' perceptions and prioritization of AI-VDH influencers versus human influencers across various presentation style dimensions.

Firstly, the ranking of presentation styles for AI-VDH influencers (Table 10) revealed that respondents placed highest importance on “natural and clear voice” (average score 5.72), followed by “vivid facial expressions” (4.74) and “coordinated and natural body movements” (4.55).

**Table 10:** Respondents' assessment of characteristics of AI-VDH influencers.

Factors	Does the voice of AI-VDH influencers sound natural and clear	Is the facial expression of AI-VDH influencers vivid and rich	Whether the eyes of AI-VDH influencers are flexible and attractive	Whether the body movements (such as gestures) of AI-VDH influencers are coordinated and natural	Whether the appearance and dress of AI-VDH influencers are decent and professional	The gender of the AI-VDH influencers	Whether the AI-VDH influencers looks like a real person
Ranking	1	2	4	3	5	7	6
Score mean	5.7228	4.7426	4.4653	4.5545	4.198	1.7723	2.5446
Score variance	1.7299	1.4048	1.2631	1.3822	1.7577	1.2258	1.9424
Ranked 1st	50 (49.5%)	11 (10.9%)	5 (5%)	8 (7.9%)	17 (16.8%)	1 (1%)	9 (8.9%)
Ranked 2nd	21 (20.8%)	23 (22.8%)	11 (10.9%)	23 (22.8%)	12 (11.9%)	5 (5%)	6 (5.9%)
Ranked 3rd	8 (7.9%)	22 (21.8%)	39 (38.6%)	17 (16.8%)	12 (11.9%)	/	3 (3%)
Ranked 4th	8 (7.9%)	25 (24.8%)	27 (26.7%)	28 (27.7%)	9 (8.9%)	/	4 (4%)
Ranked 5th	7 (6.9%)	16 (15.8%)	12 (11.9%)	19 (18.8%)	37 (36.6%)	2 (2%)	8 (7.9%)
Ranked 6th	2 (2%)	2 (2%)	4 (4%)	6 (5.9%)	12 (11.9%)	43 (42.6%)	32 (31.7%)
Ranked 7th	5 (5%)	2 (2%)	3 (3%)	/	2 (2%)	50 (49.5%)	39 (38.6%)

**Figure 7:**
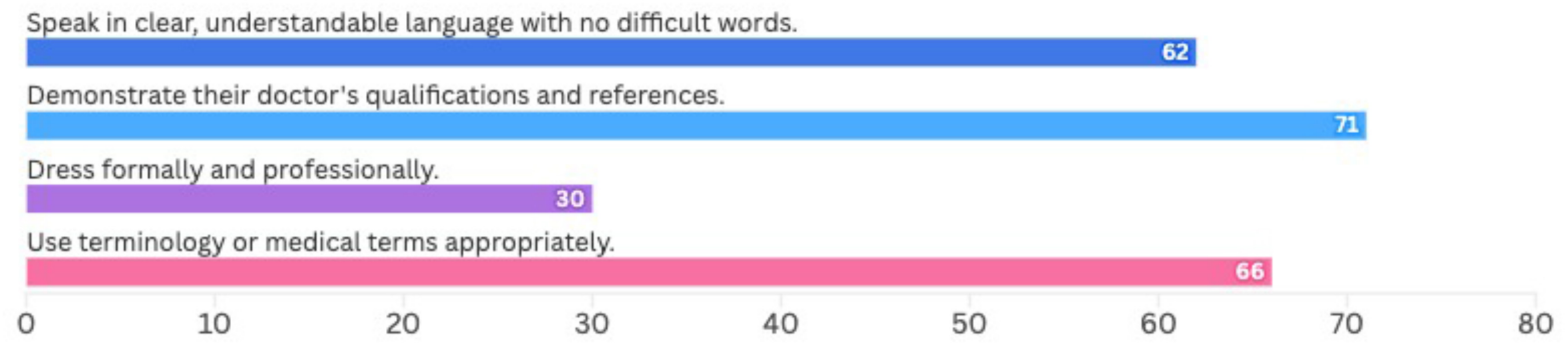
Respondents' perception of the credibility of physician influencers.

Secondly, the credibility of real-person influencers is primarily built upon identity verification and content expertise, placing greater emphasis on intuitive information cues derived from social authority or symbolic representations (Figure 7).

Comparing the presentation styles of the two types of content creators, it can be seen that older adults conduct credibility assessment mainly by initiating a heuristic processing mode, which uses concrete, perceptible, intuitive cues (e.g., voices, facial expressions, and gestures) as the core criterion rather than analyzing the information logically or the evidence deeply and systematically.

Although attitudes toward artificial intelligence vary among older adults, data indicates they place greater trust in human influencers for professionalism and persuasiveness, while maintaining heightened vigilance when identifying AI-generated content. However, when assessing the credibility of health-related short videos, older adults prioritize content practicality regardless of influencer type and avoid overly complex information. Furthermore, differences in presentation style influence trustworthiness, with the credentials of medical practitioners emerging as a key determining factor.

Comparing the presentation styles of these two influencer categories reveals that when assessing credibility, older adults primarily employ heuristic processing. This approach centers on concrete, perceptible intuitive cues—such as voice, facial expressions, and gestures—rather than deep, systematic analysis of informational logic or evidence.

Although older adults hold divergent attitudes toward artificial intelligence, data indicates they place greater trust in human influencers regarding professionalism and persuasiveness, while exhibiting heightened vigilance when identifying AI-generated content. However, in assessing the credibility of health-related short videos, older adults prioritize content practicality regardless of influencer type and avoid overly complex information. Furthermore, presentation style differences influence trust levels, with the credentials of medical practitioners serving as a key determinant.

### 4.4 Cognitive enhancement and attitudinal shift

This section focuses on RQ3, integrating qualitative interviews and quantitative data to explore the differences in the impact of AI-VDH influencers and human influencers on older adults' perceptions of health and shifts in their attitudes. In the interviews conducted as part of the experiment, multiple respondents repeatedly emphasized that when evaluating health information, they prioritize whether the content itself is logically presented and clearly explained, rather than the identity or appearance of the influencer.

“*As long as the content makes sense, I'll listen carefully, no matter if it's AI or not.” (GA6)*

Such responses indicate that when encountering health-related short videos, audiences tend to base their judgement on rational analysis of the content rather than being driven by external cues. Building on the qualitative findings described above, the quantitative results provide further supporting evidence (Table 11).

**Table 11:** Respondents' motivations for acquiring information.

Questions	Median	Mean	Standard deviation
Q12: I am very concerned about the accuracy and reliability of health information in health short videos	4	4.22	0.93
Q13: I hope to gain health knowledge from health short videos	5	4.48	0.82
Q16: I usually deeply consider the logic and scientific basis of health short video content and check its sources	4	4.23	0.91
Q17: For uncertain content, I know how to verify the accuracy of health information mentioned in short videos	4	3.95	0.99

Furthermore, regarding the differences between AI-VDH influencers and human influencers in enhancing older adults' health awareness and attitudes, the findings in Table 12 indicate that while both video formats demonstrate positive effects in improving cognition and attitudes, older adults generally respond more favorably to human influencers.

**Table 12:** Differences between AI-VDH influencers and physician influencers in enhancing health perceptions and attitudes among older adults.

Questions	Median	Mean	Standard deviation
Q27: After watching a health short video created by an AI-VDH influencer, my relevant health knowledge increased	4	4.07	0.88
Q28: After watching a health short video created by a real physician influencer, my relevant health knowledge increased	5	4.33	0.89
Q29: After watching a health short video created by an AI-VDH influencer, my attitude towards a healthy lifestyle became more positive	4	4.05	0.94
Q30: After watching a health short video created by a real physician influencer, my attitude towards a healthy lifestyle became more positive	4	4.25	0.88

From a cognitive perspective, the findings of Q27 and Q28 indicate that respondents were more inclined to believe real-person influencers could deliver more significant knowledge enhancement. This outcome may be linked to the heightened perceived credibility of real-person influencers, with respondents more readily viewing them as “authoritative figures” and consequently receiving and internalizing their messages with greater seriousness.

Regarding attitudes, while Q29 and Q30 results show human influencers still hold a slight edge, virtual characters can also stimulate older adults' attention and interest in health to a certain extent.

Both AI-VDH and doctor influencers can disseminate health knowledge and foster positive attitudes; however, in terms of cognitive enhancement and attitude shaping, human influencers slightly outperform AI counterparts, likely due to their higher perceived credibility. AI-VDH influencers also demonstrated significant positive impacts, indicating the potential of virtual characters in health communication. The older population prioritize content logic and scientific rigor over influencer status when viewing health videos, reflecting their capacity for systematic information processing and active verification.

## 5 Discussion

This study, focusing on China's older population, introduces credibility assessment theory to compare the trustworthiness of AI-VDHs and medical professionals in health-related short videos. It not only extends previous discussions on generative AI from text and conversational interfaces to embodied virtual avatars but also provides new evidence and theoretical extensions for understanding how older audiences evaluate health information within emerging media environments and new technologies.

### 5.1 Health motivation and health literacy among older adults: a reassessment in the context of AI-VDH

Compared to younger individuals, older adults exhibit a markedly practical-oriented motivation toward health information. When short video content closely aligns with daily wellness practices or chronic disease management and appears plausible, they are willing to invest cognitive resources within their experiential framework to conduct limited logical and scientific inferences regarding the content's coherence. Conversely, on topics perceived as higher-risk or requiring greater professional expertise, they tend to rely on offline medical consultations or authoritative physician judgements rather than actively cross-verifying platforms and AI-generated information like younger samples (63). This indicates that older adults' cognitive processing exhibits pronounced “topic selectivity,” with practicality and risk perception jointly defining the scope of information they are willing to “consider seriously.”

Regarding capability dimensions, older adults believe they can identify AI-generated content through visual or auditory cues and rely on heuristic judgments based on authoritative accounts, experience, and anti-fraud awareness (35); however, their actual identification accuracy is low, and they may still misjudge even when platform prompts are present (44). The “high subjective confidence—low objective recognition ability” pattern indicates that summarizing capabilities solely in terms of digital literacy, fact-checking skills, and domain knowledge is no longer sufficient (38). It is necessary to distinguish between “self-perceived ability” and “actual ability,” and to treat this discrepancy as a prerequisite that influences subsequent processing pathways (39, 64).

At the cognitive and attitudinal transformation level, research indicates that AI-VDHs have substantial persuasive potential in enhancing older adults' health knowledge and shaping their health attitudes. Experiments and questionnaires indicate that health-related short videos can significantly improve both knowledge and health attitudes; once a judgement of basic credibility is formed regarding content and source, high health motivation drives active internalization of information, which aligns with findings by Winterstein et al. (24) and Stein et al. (23) regarding the roles of AI. Although physician influencers are slightly more effective, AI-VDHs are better suited for routine, standardized health topics, while physicians still need to step in for high-risk decisions (25). This suggests that older adults' health literacy is taking on a new form: while relying on physicians for critical decisions, they maintain conditional, selective trust in AI-VDHs.

### 5.2 Contextualized refinement of the dual-process–three-level theoretical framework

At the theoretical level, this study does not seek to structurally redefine the foundational model, but rather applies and refines 38 “stage × level” integrated framework to an emerging field. By focusing on the intersection of health communication for older adults and AI technology, this study provides a contextualized refinement of the dual-processing mechanism.

At the construct level, older adults' credibility judgements primarily focused on two core constructs: firstly, the content construct, encompassing whether information was accurate, logically coherent, and closely relevant to chronic disease management and daily health maintenance; secondly, the source construct, concentrating on whether the doctor was professional and affiliated with an authoritative institution. This aligns strongly with the emphasis on content quality and authority highlighted by by Choi et al. (38) and Lawal and Stvilia (63). Experiments and questionnaires indicate that older adults predominantly employ a parallel processing pathway of “content-based systematic processing + source-identity heuristic processing.” Even when unable to determine whether content is AI-generated, they fundamentally weigh it against both content quality and authority dimensions. In contrast, homophily, relatability, rawness, audience participation, and social sharing—prominent in Chang et al.'s (35) model among younger samples—were markedly diminished in this study. Some participants even perceived these as factors undermining professional credibility (65). This indicates that within the high-risk, highly specialized context of older population health short videos, the triad of “practicality + information quality + professional authority” constitutes the “hard core” of credibility assessment, with construct weights undergoing significant rearrangement according to age and media context.

At the heuristic level, this study corroborates findings that older adults heavily rely on everyday experience and life knowledge for judgement (35, 44), typically manifested through the “self-affirmation heuristic”: regardless of whether the communicator is AI-VDH or a physician, “whether it aligns with common sense” and “whether it is logically sound” serve as primary criteria. Labels such as expert or tertiary hospital reflect reliance on the reputation heuristic; heightened vigilance toward promotional content and exaggerated therapeutic claims corresponds to the persuasive intent heuristic. In AI-VDHs scenarios, older adults further attempt to distinguish human/machine identity through experience: when appearance, tone, or movements deviate from established real doctor schemata, they tend to conclude “this is AI,” extending the application of the expectancy-violation heuristic (64). However, when AI-VDHs exhibit highly realistic appearance and expression, this heuristic fails. Older adults struggle to intuitively distinguish AI from humans, amplifying the capability mismatch characterized by high subjective confidence but low actual recognition ability. Conversely, the endorsement heuristic manifests as interpersonal/institutional recognition rather than popular recognition among older adults: their initial attitudes toward AI-VDHs are shaped more by friends' and relatives' experiences and official discourse than by platform interaction data (38). This suggests the theoretical necessity to expand endorsement from a platform data-driven mechanism to a broader social recognition framework encompassing interpersonal and institutional endorsements.

At the cue level, this study identifies author credentials and content type as the most influential cues, while engagement metrics such as like counts, comment volumes, follower numbers, and cross-platform activity are scarcely mentioned proactively among older adult participants (38, 63). This indicates a distinct shift in the cues layer from interaction data-driven to credentials and content-driven within the context of health-related short videos for older adults. Within AI-VDHs scenarios, respondents noted clear, natural voices and standardized terminology. However, their focus on whether facial expressions, movements, and appearance resembled real people primarily served identity verification rather than directly enhancing credibility. Instead, authoritative cues—such as displaying medical credentials and employing professional terminology—proved pivotal in influencing credibility judgements. Health-themed proximity cues, meanwhile, increase older adults' willingness to invest cognitive resources in content evaluation (35). Furthermore, this study identifies platform-level AI labeling cues that Choi et al. (38) and Lawal and Stvilia (63) did not sufficiently emphasize: many older adults actively notice system labels such as “AI-generated” or “suspected AI” and accordingly lower or recalibrate their expectations regarding video credibility. This demonstrates that platform labels have become significant cues triggering heuristic processing.

In summary, while retaining the fundamental structure of 38 dual-processing–three-level model, this study identifies three contextual elaborations specific to the domain of older population health communication and AI-VDHs. Firstly, within the capability dimension of the exposure stage, it is found that distinguishing between “subjective sense of capability” and “objective recognition ability”—and the potential discrepancy between them—serves as a contextualized precondition influencing path selection. Second, during the evaluation phase, this research illustrates a composite pathway where systematic and heuristic processing co-operate via rational content assessment and source identity shortcuts, rather than as mutually exclusive alternatives. Third, at the cue level, the study highlights the necessity of integrating platform AI identifiers and institutional endorsements within a unified framework, thereby specifying the role of intelligent media cues. Consequently, this study provides a more nuanced delineation of the model's applicability boundaries and extension directions within embodied AI contexts for older adults.

### 5.3 Practical implications and recommendations

Firstly, in practice, the design of relevant products and platforms should better align with the cognitive processing characteristics of older adults, rather than merely pursuing technological realism. Specifically, rather than continuing to invest substantial resources in making virtual avatars more lifelike, priority should be given to ensuring the scientific accuracy, logical coherence, and practical applicability of the content itself. This would enable it to adequately support the limited yet genuinely existing systematic processing capabilities of older adults, particularly when driven by high health motivation. Concurrently, interfaces and narratives should clearly display physician credentials, institutional affiliations, and conspicuous, legible “AI-generated” disclosures. This ensures critical credibility cues can be efficiently accessed through heuristic processing by older adults, rather than being obscured by algorithmic manipulation or visual packaging.

Conversely, this study also highlights potential ethical and regulatory risks associated with AI-VDH: should virtual avatars be employed to feign authority, exaggerate therapeutic efficacy, or steer excessive consumption, they may amplify the impact of misinformation and commercial deception within a “high confidence-low discernment” framework. Consequently, subsequent theories, policies, and industry standards concerning AI health communication should, while acknowledging its scalability advantages in disseminating health knowledge, incorporate “preventing the exploitation of AI avatars to manipulate vulnerable groups” trust' as a necessary constraint within the dual-processing framework of health communication. This requires strengthening platform review responsibilities and cultivating digital and AI literacy among the older population.

### 5.4 Limitations

First, the generalizability of this study's findings is somewhat limited. Since the eligibility criteria required participants to have prior experience watching AI-generated short videos, the sample naturally skews toward older adults with higher digital literacy. Consequently, the observed cognitive patterns may not fully represent the broader Chinese older adult population, particularly those living in areas with limited digital connectivity or in rural regions. Second, this study relies on self-report scales and interview statements, which may introduce self-report bias. Participants may have provided socially desirable responses or found it difficult to accurately recall their cognitive load and emotional changes. Finally, as an exploratory study, the small sample size (*n* = 18) limits the statistical power for broad theoretical generalizations. Although these findings offer valuable contextual refinements to the dual-processing framework, they should be considered preliminary conclusions. Future research should employ more diverse sampling frames, incorporating objective physiological measures such as eye-tracking or EEG to provide granular data on real-time information processing and further validate the structural stability of the proposed theoretical extensions.

## 6 Conclusion

This study examines sampled Chinese seniors' early experiences with health-related short videos, comparing AI-VDH influencers with physician influencers across discernibility, credibility assessment, and health cognition/attitude promotion. Findings reveal that while the participating seniors subjectively express confidence in distinguishing “AI from real persons,” their actual recognition accuracy remains limited under highly realistic mixed stimuli, exhibiting a classic “high confidence-low competence” mismatch. Regarding credibility assessment, they do not choose between heuristic and systematic processing, but instead employ limited systematic processing based on “the scientific validity and practical applicability of content,” while simultaneously relying on source identity cues such as medical titles and hospital affiliations for heuristic verification. Physicians demonstrate overall superior credibility and slightly better knowledge and attitude promotion effects than AI-VDHs among the study participants. However, the latter also exhibit stable communication efficacy in low-risk, standardized topics such as wellness and chronic disease management. Further findings reveal that these older adults' initial attitudes toward AI-VDHs are predominantly shaped by social endorsements derived from personal/acquaintance experiences and official institutions. Concurrently, platform interface labels such as “AI-generated” serve as crucial cues for adjusting trust expectations, indicating that system labels themselves have become novel triggers for heuristic processing. Building upon these findings, this paper proposes a preliminary contextual extension of traditional dual-processing model of credibility by introducing a contextualized framework: exploring capability mismatch as the potential prerequisite linking exposure and evaluation stages. It suggests that older adults in this context do not consistently follow central or peripheral processing pathways, but rather dynamically switch between systematic and heuristic processing based on health motivation, topic risk, cognitive load, and platform cues. In most observed scenarios, both pathways operate concurrently, forming a composite model of “content-rational evaluation + source-identity shortcut”. Furthermore, the study defines AI-VDHs as a “technological communicator” possessing scalable advantages in low-risk utilitarian topics while requiring human experts to fill gaps in high-risk contexts. This offers preliminary insights into how AI-generated virtual health communicators embed themselves within older adults' trust structures. Finally, the evidence for recognition and persuasion effects in this study primarily stems from self-reported scales and interview statements. While subsequent research should employ more diverse methodology and longitudinal designs to further validate these mechanism chains, this study provides a foundational understanding of AI-driven health communication for older adults. By identifying the “calibration bias → cue dependence” logic, this research underscores the urgency for public health authorities and platform providers to develop age-appropriate AI transparency standards. Enhancing the digital health literacy of the older population while ensuring the ethical use of AI influencers will be pivotal in fostering a trustworthy intelligent media environment for a rapidly aging society.

## Data Availability

The original contributions presented in the study are included in the article/Supplementary material, further inquiries can be directed to the corresponding author.
