# Supplementary material for: AI virtual digital human influencers vs. human influencers: the impact of health short videos on older adults' cognition and attitude
Source: Front Public Health. 2026 Jun 4;14:1805541. doi: 10.3389/fpubh.2026.1805541 (PMC13275421; doi:10.3389/fpubh.2026.1805541)
Supplement: Supplementary file 1 [file Supplementary_file_1.docx]

**Appendix 1: Pre-test Questions**

1. Name:
2. Age:
3. Gender:

□ Male □ Female

1. Education Level:

□ Primary school and below □ Junior high school □ High school □ University and above

1. Do you live alone?

□ Yes □ No

1. Economic Status (Monthly household income):

□ < ¥4,000 □ ¥4,001 - ¥6,000 □ ¥6,001 - ¥9,000 □ > ¥9,001

1. How often do you watch short videos?

□ > 2 hours per day □ 1-2 hours per day □ 0.5-1 hour per day □ < 0.5 hours per day

1. What types of health information do you usually pay attention to? [Multiple Choice]

□ Medical: Includes content on prevention and treatment of cancer, chronic diseases, cardiovascular diseases, and orthopedic knowledge for the elderly.

□ Wellness: Includes content on exercise, health-preserving recipes, health, and disease prevention.

□ Health News & Information: Includes information on epidemics, prevention measures, and vaccination.

1. What are your main channels for getting health information? [Multiple Choice]

□ WeChat Channels □ Douyin □ Kuaishou □ Friends and family sharing □ Doctor

□ Television □ Radio □ Books and newspapers □ Other: ______

1. What elements must a health short video have for you to find it credible? (e.g., content, influencer's identity, voice, attire, tone, whether it's platform-certified, number of likes and comments, etc.)
2. Are you familiar with AI technology and AI virtual digital humans? Are you aware that some characters in short videos are not real people?
3. Have you noticed the "Likely generated using AI technology, please exercise caution." label on short video platforms? Does this label affect your judgment of the influencer's authenticity?
4. If an AI virtual digital human talks about health knowledge, compared to a real physician influencer, who do you think is more trustworthy? Why?

**Appendix 2 Focus Group Interview Questions**

1. Among the short videos you just watched, which information was different from what you already knew?
2. What aspects of the influencers caught your attention?
3. When you were watching the short videos, were you able to distinguish whether the influencer was a real person or an AI virtual digital human? If yes, what characteristics helped you make that judgment (e.g., voice, tone, naturalness of movements, facial expressions)?
4. In your opinion, what are the differences between an AI virtual digital human influencer and a real physician influencer in terms of language expression (e.g., word choice, speech speed) and movement design? Are these differences obvious?
5. Compared to a real physician influencer, does the voice of an AI virtual digital human influencer (e.g., synthetic sound, mechanical feel, emotional richness, speed of speech, coherence) make you more trusting or more suspicious of their health advice? Why?
6. Do you find the lifelike design of the AI virtual digital human influencer's image to be professional or unreliable? Please give an example.
7. Whose health advice do you find more convincing: a physician influencer's or an AI virtual digital human influencer's? Why?
8. Would you change your health habits based on the advice from a physician influencer or an AI virtual digital human influencer? Why?
9. Are you willing to share these health short videos with family or friends? Does your willingness to share depend on the type of influencer or the health content?

**Appendix 3 Delayed Post-Test Questions**

1. Have you recently come across any AI-generated short videos?
2. When you watch short videos now, can you more clearly distinguish between a real influencer and an AI virtual digital human influencer?
3. Compared to the beginning, has your trust in the content from AI virtual digital human influencers changed? Why or why not?
4. If you see a video of an AI virtual digital human influencer talking about health knowledge in the future, would you trust the content they are sharing?
5. Are you now willing to forward a health short video from an AI virtual digital human influencer to others? Why or why not?

**Appendix 4 Questioner on The Impact of Health Short Videos on Older Adults’ Cognition and Attitude between AI Virtual Digital Human Influencers vs Human Influencers**

Dear Participant,

Hello! We are conducting a study on health short videos on social media. This survey aims to understand your opinions on health short videos narrated by " AI Virtual Digital Human Influencers" (virtual influencers generated by artificial intelligence) and "Physician Influencers," and how these videos influence your judgment of health information, as well as your health cognition and attitude. Your responses are crucial to our research, and all information will be used for academic purposes only. Your personal information will be kept strictly confidential. Thank you for your support and cooperation!

**Section I: Basic Information**

1. **Your age** [Single Choice]

- Below 60 years old
- 60-69 years old
- 70-79 years old
- 80 years old and above

1. **Your gender** [Single Choice]

- Male
- Female

1. **Your education level** [Single Choice]

- Primary school and below
- Junior high school
- High school / Vocational school
- College (Associate's Degree)
- Bachelor's degree and above

1. **Your current residence type** [Single Choice]

- City
- Town / Small town
- Rural area

1. **Do you live alone?** [Single Choice]

- Yes
- No

1. **Your total monthly household income is approximately** [Single Choice]

- Below ¥3,000
- ¥3,000–¥4,999
- ¥5,000–¥7,999
- ¥8,000–¥9,999
- ¥10,000 and above
- Prefer not to say

1. **Do you frequently use the following platforms to watch health short videos?** [Multiple Choice]

- Douyin
- Kuaishou
- Xigua Video
- WeChat Channels
- Toutiao
- I do not use them
- Others: ______

1. **Approximately how much time do you spend watching short videos per day?** [Single Choice]

- Less than 30 minutes
- 30 minutes–1 hour
- 1–2 hours
- More than 2 hours

**Section II: Main Questionnaire**

Instructions: Please read each question carefully and select the number that best reflects your true feelings.

Scale Options: 1 = Strongly Disagree, 2 = Disagree, 3 = Neutral, 4 = Agree, 5 = Strongly Agree

**When you have watched health short videos in the past:**

1. **In the health short videos, I have watched that were produced by AI Virtue Digital Human Influencers, I think** [Multiple Choice]

- Their language is clear and fluent.
- Their facial expressions are natural and not stiff.
- Their eyes are lively and expressive.
- Their body movements are coordinated and natural, and their postures are varied.
- Their appearance is indistinguishable from that of real people.
- Their content is easy to understand and has a certain level of knowledge.
- None of the above.

1. **In the health short videos, I have watched that were produced by physician influencers, I think** [Multiple Choice]

- Their language is clear and fluent.
- Their facial expressions are natural and not stiff.
- Their eyes are lively and expressive.
- Their body movements are coordinated and natural, and their postures are varied.
- Their look professional and reliable.
- Their content is easy to understand and has a certain level of knowledge.
- None of the above.

1. **The types of health short videos I often watch are** [Multiple Choice]

- Medical: including content on the prevention and treatment of cancer, chronic diseases, cardiovascular diseases, and orthopedic knowledge for the elderly.
- Wellness: including content on exercise, health-preserving recipes, health and disease prevention.
- Health news and information: including information on epidemics, prevention measures, and vaccination.
- I watch almost anything, as long as the content in the video is beneficial to my family and friends.
- I watch almost none.

1. **I am very concerned about the accuracy and reliability of health information in videos.** [Scale Question]

Strongly Disagree | Strongly Agree

1 | 2 | 3 | 4 | 5

1. **I hope to gain health knowledge from health short videos.** [Scale Question]

Strongly Disagree | Strongly Agree

1 | 2 | 3 | 4 | 5

**When evaluating health short videos on social media:**

1. **I typically rely on my own experience and knowledge to quickly judge the credibility of health information.** [Scale Question]

Strongly Disagree | Strongly Agree

1 | 2 | 3 | 4 | 5

1. **I often quickly assess the credibility of health short video content based on the influencer's superficial image or the number of likes/comments.** [Scale Question]

Strongly Disagree | Strongly Agree

1 | 2 | 3 | 4 | 5

1. **I usually deeply consider the logic and scientific basis of video content and check its sources.** [Scale Question]

Strongly Disagree | Strongly Agree

1 | 2 | 3 | 4 | 5

1. **For uncertain content, I know how to verify the accuracy of health information mentioned in health short videos.** [Scale Question]

Strongly Disagree | Strongly Agree

1 | 2 | 3 | 4 | 5

1. **I usually don't specifically evaluate the accuracy of health content in health short videos.** [Scale Question]

Strongly Disagree | Strongly Agree

1 | 2 | 3 | 4 | 5

1. **I am confident in distinguishing between AI Virtue Digital Human influencers and human influencers in health short videos.** [Scale Question]

Strongly Disagree | Strongly Agree

1 | 2 | 3 | 4 | 5

1. **I can usually easily tell whether a health short video influencer is an AI Virtue Digital Human influencer or a human influencer.** [Scale Question]

Strongly Disagree | Strongly Agree

1 | 2 | 3 | 4 | 5

1. **I usually notice the "Potentially AI-generated content, please exercise caution" disclaimer below some health short videos.** [Scale Question]

Strongly Disagree | Strongly Agree

1 | 2 | 3 | 4 | 5

1. **If the influencer is a physician from a certain hospital, I will trust their health advice because they _____** [Multiple Choice]

- Speak in clear, understandable language with no difficult words.
- Demonstrate their doctor's qualifications and references.
- Dress formally and professionally.
- Use terminology or medical terms appropriately.

1. **If an influencer is an AI Virtue Digital Human, I will be more cautious when evaluating health information.** [Scale Question]

Strongly Disagree | Strongly Agree

1 | 2 | 3 | 4 | 5

1. **I believe real physician influencers provide health knowledge that is more professional and useful than AI Virtue Digital Human influencers.** [Scale Question]

Strongly Disagree | Strongly Agree

1 | 2 | 3 | 4 | 5

1. **I believe real human influencers provide health knowledge that is more professional and useful than AI Virtue Digital Human influencers.** [Scale Question]

Strongly Disagree | Strongly Agree

1 | 2 | 3 | 4 | 5

1. **Which of the following AI Virtue Digital Human Influencer characteristics most influences your acceptance of the health information in their health short videos? Please rank them by influence level.** [Ranking Question]

- Does the voice of AI Virtue Digital Human Influencer sound natural and clear?
- Is the facial expression of AI Virtue Digital Human Influencer vivid and rich.
- Whether the eyes of AI Virtue Digital Human Influencer are flexible and attractive.
- Whether the body movements (such as gestures) of AI Virtue Digital Human Influencer are coordinated and natural.
- Whether the appearance and dress of AI Virtue Digital Human Influencer is decent and professional.
- The gender of the AI Virtue Digital Human Influencer.
- Whether the AI Virtue Digital Human Influencer looks like a real person.

**After watching a health short video on social media, I think:**

1. **After watching a health short video created by an AI Virtue Digital Human influencer, my relevant health knowledge increased**. [Scale Question]

Strongly Disagree | Strongly Agree

1 | 2 | 3 | 4 | 5

1. **After watching a health short video created by a real human influencer, my relevant health knowledge increased.** [Scale Question]

Strongly Disagree | Strongly Agree

1 | 2 | 3 | 4 | 5

1. **After watching a health short video created by an AI Virtue Digital Human influencer, my attitude towards a healthy lifestyle became more positive.** [Scale Question]

Strongly Disagree | Strongly Agree

1 | 2 | 3 | 4 | 5

1. **After watching a health short video created by a real human influencer, my attitude towards a healthy lifestyle became more positive.** [Scale Question]

Strongly Disagree | Strongly Agree

1 | 2 | 3 | 4 | 5

1. **I would adjust my diet, exercise, or medication habits based on the content of a health short video.** [Scale Question]

Strongly Disagree | Strongly Agree

1 | 2 | 3 | 4 | 5

1. **I am willing to share health short videos that I consider credible with my friends and family.** [Scale Question]

Strongly Disagree | Strongly Agree

1 | 2 | 3 | 4 | 5

♥ Thank you for completing this survey!
